# Meta-analysis of outcomes following intravenous thrombolysis in patients with ischemic stroke on direct oral anticoagulants

**DOI:** 10.1186/s12883-023-03498-8

**Published:** 2023-12-15

**Authors:** Amir Hossein Behnoush, Amirmohammad Khalaji, Pegah Bahiraie, Rahul Gupta

**Affiliations:** 1 School of Medicine, Tehran University of Medical Sciences, Poursina St., Keshavarz Blvd, Tehran, 1417613151 Iran; 2 Cardiovascular Diseases Research Institute, Tehran Heart Center, Tehran University of Medical Sciences, Tehran, Iran; 3 School of Medicine, Shahid Beheshti University of Medical Sciences, Tehran, Iran; 4 Department of Cardiology, Lehigh Valley Health Network, Allentown, PA USA

**Keywords:** Intravenous thrombolysis, Stroke, Factor xa inhibitors, Systematic review, Meta-analysis

## Abstract

**Background:**

There has been debate on the use of intravenous thrombolysis (IVT) in patients with ischemic stroke and the recent use of direct oral anticoagulants (DOACs). Studies have compared these patients with non-DOAC groups in terms of outcomes. Herein, we aimed to systematically investigate the association between DOAC use and IVT’s efficacy and safety outcomes.

**Results:**

A comprehensive systematic search was performed in PubMed, Embase, Scopus, and the Web of Science for the identification of relevant studies. After screening and data extraction, a random-effect meta-analysis was performed to calculate the odds ratio (OR) and 95% confidence interval (CI) for comparison of outcomes between patients on DOAC and controls. Six studies were included in the final review. They investigated a total of 254,742 patients, among which 3,499 had recent use of DOACs. The most commonly used DOACs were rivaroxaban and apixaban. The patients on DOAC had significantly higher rates of atrial fibrillation, hypertension, diabetes, and smoking. Good functional outcome defined by modified Rankin Scale (mRS) 0–2 was significantly lower in patients who received DOACs (OR 0.71, 95% CI 0.62 to 0.81, *P* < 0.01). However, in the subgroup analysis of 90-day mRS 0–2, there was no significant difference between groups (OR 0.71, 95% 0.46 to 1.11, *P* = 0.14). All-cause mortality was not different between the groups (OR 1.02, 95% CI 0.68 to 1.52, *P* = 0.93). Similarly, there was no significant difference in either of the in-hospital and 90-day mortality subgroups. Regarding symptomatic intracranial hemorrhage (sICH), the previous DOAC use was not associated with an increased risk of bleeding (OR 0.98, 95% CI 0.69 to 1.39, *P* = 0.92). A similar finding was observed for the meta-analysis of any ICH (OR 1.15, 95% CI 0.94 to 1.40, *P* = 0.18).

**Conclusions:**

Based on our findings, IVT could be considered as a treatment option in ischemic stroke patients with recent use of DOACs since it was not associated with an increased risk of sICH, as suggested by earlier studies. Further larger studies are needed to confirm these findings and establish the safety of IVT in patients on DOAC.

**Supplementary Information:**

The online version contains supplementary material available at 10.1186/s12883-023-03498-8.

## Background

Stroke is among the leading causes of death and morbidity worldwide which accounted for 12.2 million incident cases based on the Global Burden of Disease 2019 with ischemic stroke accounting for 62.4% of all stroke cases [1]. Atrial fibrillation (AF) can be considered the major risk factor for ischemic stroke that can cause extensive cerebral lesions and long-term neurological damage for which anticoagulants are indicated [2, 3]. While vitamin-K antagonists (VKAs) used to be the main anticoagulation strategy in patients with AF, direct oral anticoagulants (DOACs) have emerged as the preventive medication for stroke in patients with non-valvular AF [4]. Almost 1–2% of patients on DOAC for AF experience ischemic stroke yearly [5, 6].

The indications for DOAC therapy are increasing day by day, it is estimated that one in six patients undergoing intravenous thrombolysis (IVT) for ischemic stroke has a prescription of DOACs [7]. While patients on VKAs are suggested to be excluded from IVT if the international normalized ratio (INR) is greater than 1.7, patients with DOACs use within the previous 48 h are also suggested not to undergo IVT based on the presumption of increased risk of symptomatic intracranial hemorrhage (sICH) [8, 9]. However, this presumption has been challenged in a meta-analysis showing approximately 50% decreased risk of ICH in the DOAC group compared to warfarin [4, 10, 11].

DOACs seem to be different from VKAs due to the fact that, unlike VKAs, they did not increase the risk of hemorrhage after IVT in experimental studies [12–14]. Studies have been conducted in order to determine the efficacy and safety of IVT in patients with recent use of DOACs while the overall benefits and harms of it have not been elucidated yet, Herein, we aim to systematically investigate the overall effect of DOAC use in outcomes of patients with ischemic stroke undergoing IVT.

## Methods

The methods and results of this study were reported in accordance with the Preferred Reporting Items for Systematic Reviews and Meta-Analyses statement (PRISMA) [15]. The PRISMA checklist is available as Supplementary Table 1; however, the protocol of this review is not registered.

### Search strategy

A systematic search was performed in online databases including PubMed, Scopus, Embase, and the Web of Science from inception until November 27, 2023. The search included (“Cerebrovascular Disorders” OR “Stroke” OR “transient ischemic attack”) AND (“thrombolytic therapy” OR “intravascular thrombolysis” OR “fibrinolysis”) AND (“Direct Acting Oral Anticoagulant” OR “DOAC” OR “NOAC”) using Mesh and non-Mesh terms described in detail in Supplementary Table 2.

### Inclusion/exclusion criteria and screening

The studies were included and excluded based on pre-defined eligibility criteria. Inclusion criteria were the retrospective or prospective cohorts assessing the outcomes following IVT in patients with ischemic stroke and recent use of DOACs and comparing it with non-DOAC user controls. Case reports, case series, letters, conference abstracts, review articles, and studies without a control group were excluded. The screening was performed by two independent authors (AHB and AK) first using title and abstract then by full-text. Any disagreement was resolved through discussion by a third author (PB).

### Extraction and quality assessment

Included studies’ data were extracted by two independent authors using a data extraction sheet designed by a third author. The following data were extracted: (1) first author name, (2) year and country of publication, (3) the population investigated (inclusion criteria), (4) sample size and number of participants in each group, (5) mean age of each group, (6) male percentage in each group, (7) comorbidities (AF, hypertension, diabetes, hyperlipidemia, and smoking) rate in each group, and (8) the event rate of outcomes in each group. Efficacy outcomes included mortality (in-hospital and 90-days), good functional outcome (modified Rankin Scale [mRS] 0–2) (at discharge and 90-day), and mRS 0–1 (at discharge and 90-day). Also, the safety outcome was any ICH and symptomatic ICH.

Quality assessment was performed based on the Newcastle-Ottawa Scale [16] designed for cohort studies. It includes three domains, namely study selection, comparability, and outcome. The scores of 6–9 are rated as high-quality, 3–5 scores represent fair quality, and 0–2 show poor quality. Two independent authors assessed the qualities of the included studies individually and resolved any possible disagreement by discussion with a third author.

### Statistical analysis

All data analyses were performed using STATA (version 17.0, Stata Corp). Random-effect meta-analysis (DerSimonian-Laird) was conducted to calculate the odds ratio (OR) and 95% confidence interval (CI) for pooling the effects obtained by each study. Statistical heterogeneity was evaluated by Higgins’ I-square test based on Cochrane’s *Q*. The thresholds used for heterogeneity (*I*^*2*^) were ≤ 25%, 26–75%, and ≥ 75% for low, moderate, and high heterogeneity, respectively. Subgroup analysis based on the in-hospital or 90-day outcomes was also performed when possible. Finally, an assessment of publication bias was performed based on visual inspection of funnel plots in addition to Begg’s and Egger’s statistical tests [17, 18].

## Results

### Literature search, study selection, and included studies characteristics

**Fig. 1:**
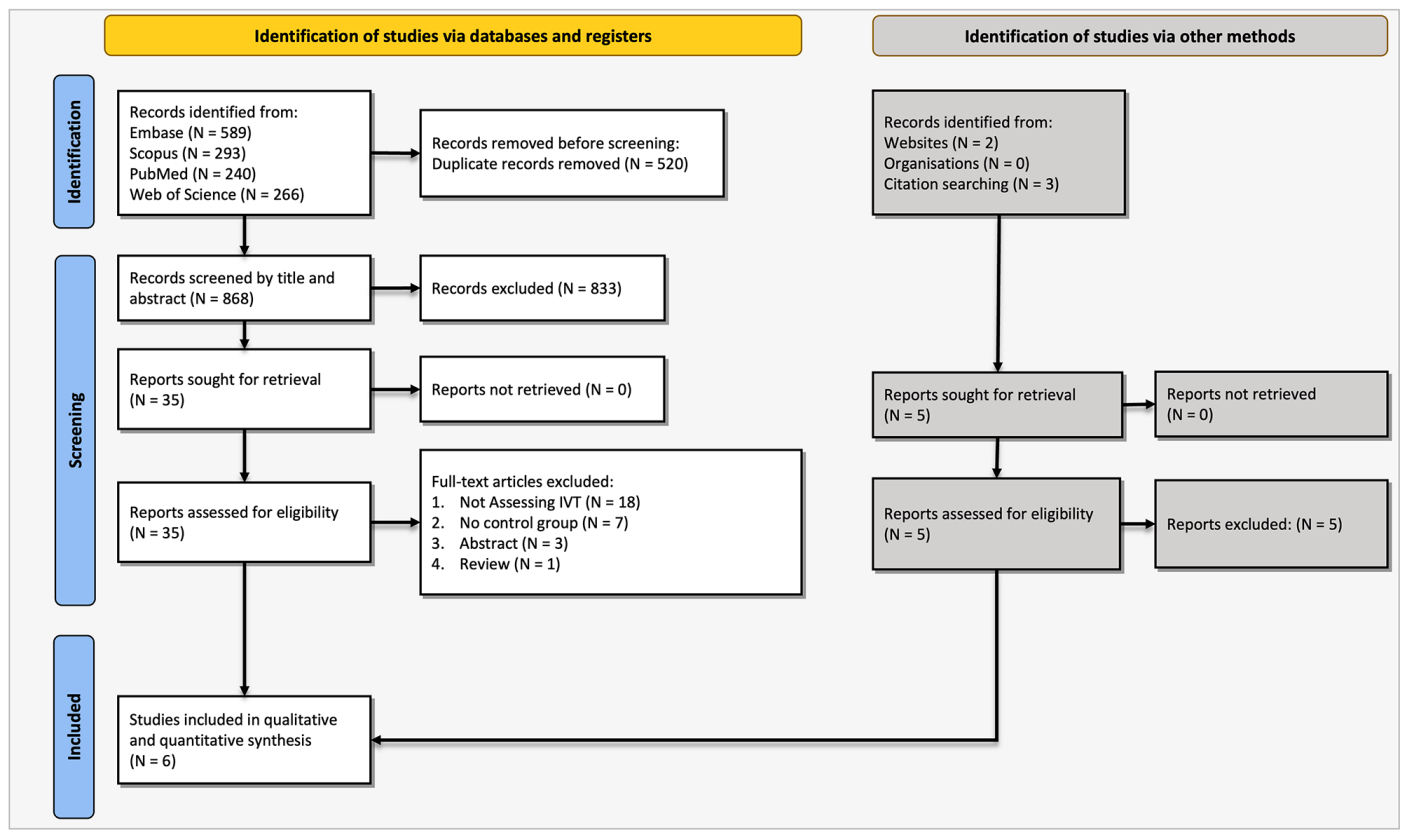
PRISMA flowchart representing the study selection process and reasons for exclusion

The initial search in the four databases yielded 1,388 results of which 520 were duplicates: 240 from PubMed, 293 from Scopus, 589 from Embase, and 266 from the Web of Science. After screening based on titles and abstracts, 35 studies remained to be assessed for full text. Finally, six studies remained to be included in the meta-analysis [7, 19–23]. The selection process and the reasons for exclusion are shown in Fig. 1.

The baseline characteristics of the six included studies are described in Table 1. Two studies were conducted in the United States [7, 22], while one was conducted in the Japan [20], one in the Europe [21], one in the Taiwan [23], and one in the multiple countries [19]. All studies were retrospective cohorts in terms of design, except one which was prospective [21]. The study by Seiffge et al. [21] compared patients with and without DOAC use, some of whom underwent IVT. Although the baseline characteristics are reported for the whole population, the outcome meta-analysis was used for IVT patients only. Among the DOACs, rivaroxaban and apixaban were included in all six studies. In comparison, dabigatran was assessed in five studies [19–23] and edoxaban was used by the patients in four studies [7, 19, 20, 23]. The qualities of the included studies were all high based on the NOS score (Supplementary Table 3).

**Table 1:** Baseline characteristics of included studies

Study	Year	Design	Country	Population	DOACs type	N total	N DOAC	N Control
**Kam et al.**	2022	Retrospective Cohort	United States	Patients with acute ischemic stroke undergoing IVT with alteplase, either taking NOACs or not taking anticoagulants	Rivaroxaban, Apixaban, or Edoxaban	163,038	2,207	160,831
**Meinel et al.**	2023	Retrospective Cohort	Europe, Asia, Australia, and New Zealand	Adult patients with ischemic stroke who underwent IVT, either with or without recent use of DOACs	Rivaroxaban, Dabigatran, Apixaban, or Edoxaban	33,207	832	32,375
**Okada et al.**	2022	Prospective Cohort	Japan	Acute ischemic stroke patients who underwent IVT with alteplase, patients with or without use of DOACs in latest 48 h	Rivaroxaban, Dabigatran, Apixaban, or Edoxaban	793	40	753
**Seiffge et al.**	2015	Retrospective Cohort	Europe	Patients with acute ischemic stroke who underwent IVT, IAT, or both	Rivaroxaban, Apixaban, or Dabigatran	9,016	78	8,938
**Xian et al.**	2017	Retrospective Cohort	United States	Patients with acute ischemic stroke who received thrombolytic therapy, with NOACs or no anticoagulation	Rivaroxaban, Apixaban, or Dabigatran	41,387	251	41,136
**Tasi et al.**	2023	Retrospective Cohort	Taiwan	Adult patients ≥ 20 years diagnosed with acute ischemic stroke treated with alteplase, with treatment status of NOAC and no oral anticoagulants	Rivaroxaban, Dabigatran, Apixaban, or Edoxaban	7,301	91	7,210

DOAC: direct oral anticoagulant, NOAC: novel oral anticoagulant, IVT: intravenous thrombolysis, IAT: intra-arterial treatment

**Fig. 2:**
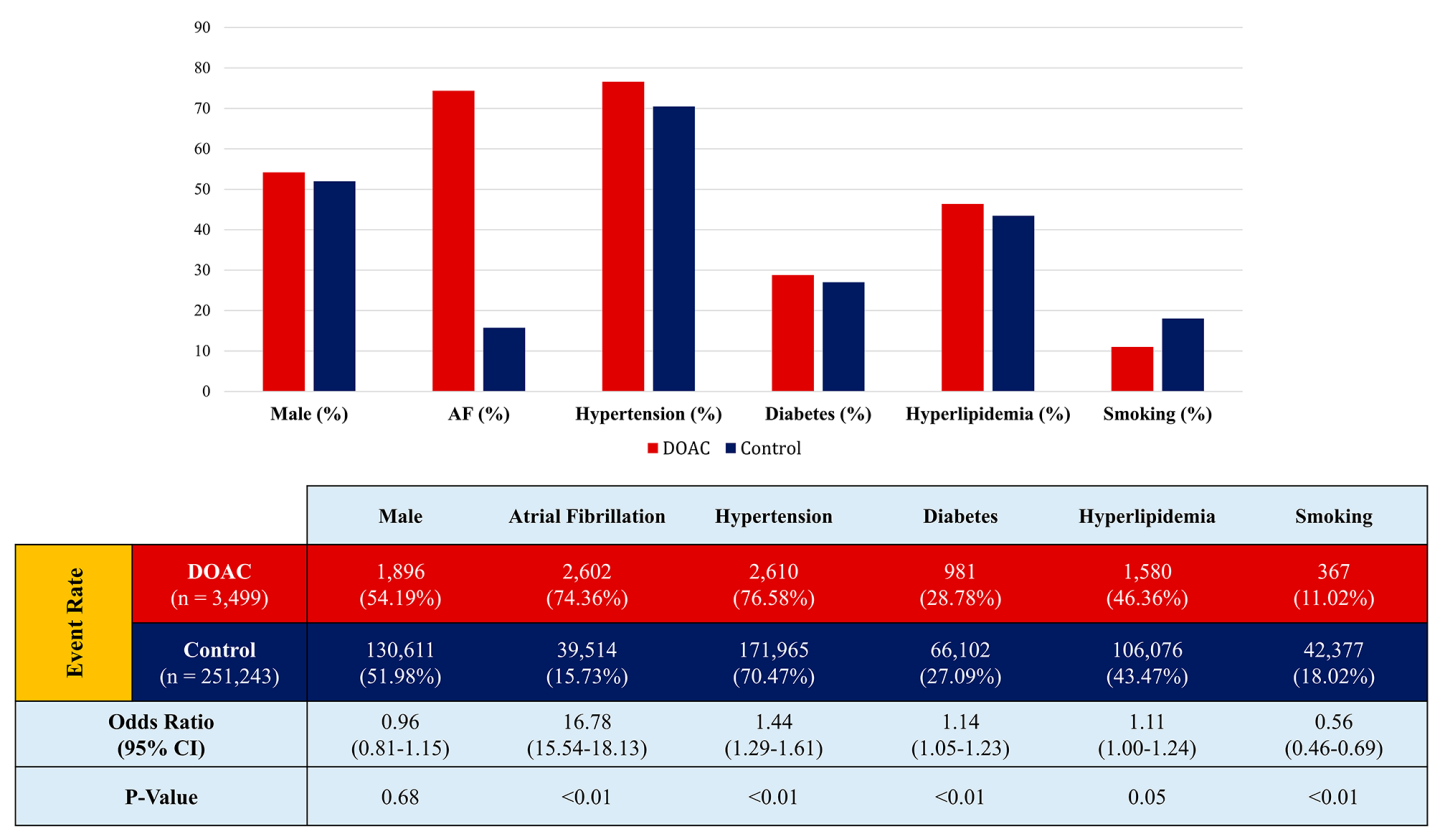
Baseline characteristics of studies’ populations

The characteristics of the included study population are shown in Table 2. A total of 254,742 patients were assessed in these studies among which 3,499 had recent use of DOACs. The mean age of patients was 74.87 ± 12.40 years in the DOAC group which was significantly higher than controls with a mean age of 69.98 ± 16.39 years (*P* < 0.01). Males contributed to 54.19% of the DOAC group and 51.98% of the non-DOAC group. As illustrated in Fig. 2, patients on DOACs had a higher rate of AF, compared with non-DOAC users (74.36% vs. 15.73%). Moreover, patients under DOAC therapy had significantly higher rates of hypertension and diabetes while a lower rate of smoking.

**Table 2:** Baseline characteristics of included studies populations

Study	Age		Male		AF		NIHSS		Hypertension		Diabetes		Hyperlipidemia		Smoking	
	DOAC	Control	DOAC	Control	DOAC	Control	DOAC	Control	DOAC	Control	DOAC	Control	DOAC	Control	DOAC	Control
**Kam et al. (2022)**	75 [64–82]	70 [58–81]	1,186 (53.7)	81,857 (50.9)	1614 (73.1)	23,458 (14.6)	10 [5–17]	7 [4–14]	1753 (79.4)	115,623 (71.9)	719 (32.6)	46,926 (29.2)	1,099 (49.8)	72,112 (44.8)	242 (11.0)	29,045 (18.1)
**Meinel et al. (2023)**	79 [71–85]	72 [62–80]	477 (57.3)	18 264 (56.4)	608 (90.1)	4,008 (25.1)	11 [6–17]	9 [5–16]	565 (75.1)	20,072 (62.2)	173 (23.2)	6,311 (19.6)	322 (43.2)	12,091 (37.6)	95 (12.8)	5,796 (19.6)
**Okada et al. (2022)**	80 [74–87]	76 [68–84]	29 (72.5)	474 (62.9)	36 (90.0)	236 (31.3)	15 [5–24]	9 [4–17]	33 (82.5)	537 (71.3)	8 (20.0)	154 (20.5)	21 (52.5)	398 (52.9)	11 (27.5)	197 (26.2)
**Seiffge et al. (2015)**	76 [68–81]	71 [60–79]	42 (53.8)	5,023 (56.2)	68 (87.2)	2,152 (24.3)	14.5 [7–19]	10 [6–16]	61 (87.1)	5,627 (63.2)	17 (24.3)	1,500 (16.8)	32 (49.2)	3,559 (41.4)	NR	NR
**Xian et al. (2017)**	74 [66–82]	71 [59–82]	122 (48.6)	20,545 (49.9)	196 (78.1)	7,430 (18.1)	12 [6–18]	9 [5–15]	198 (78.9)	30,106 (73.2)	64 (25.5)	11,211 (27.3)	106 (42.2)	17,916 (43.6)	19 (7.6)	7,339 (17.8)
**Tsai et al. (2023)**	74.1 [8.9]	67.2 [12.8]	40 (44.0)	4448 (61.7)	80 (87.9)	2230 (30.9)	14.2 [4.7]	13.0 [4.6]	NR	NR	NR	NR	NR	NR	NR	NR

Data are represented as median [IQR], mean [SD], or number (percentage)

DOAC: direct oral anticoagulants, AF: atrial fibrillation, NIHSS: National Institutes of Health stroke scale, NR: not reported

### Meta-analysis of efficacy outcomes

**Fig. 3:**
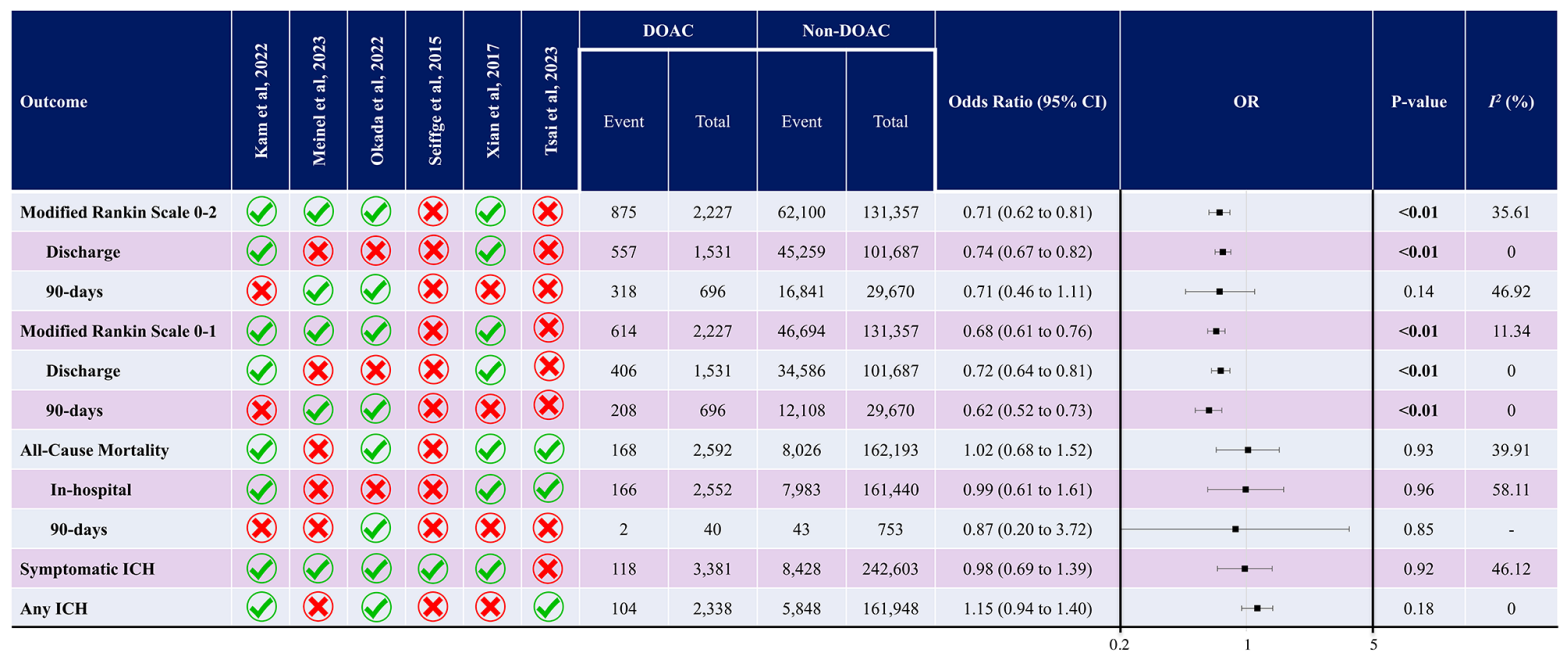
Summary of all meta-analyses regarding all-cause mortality, modified Rankin Scale 0–2, and symptomatic ICH

As shown in Fig. 3, the neurological function assessment outcome defined by the mRS 0–2 was significantly lower in patients on DOACs (OR 0.71, 95% CI 0.62 to 0.81, *P* < 0.01, Supplementary Fig. 1). This analysis was associated with 35% heterogeneity. When assessing the discharge neurological function, the DOAC group had significantly lower rates of mRS 0–2 (OR 0.74, 95% CI 0.67 to 0.82, *P* < 0.01, *I*^*2*^: 0%). However, in 90-day mRS 0–2, there was no significant difference between the groups (OR 0.71, 95% CI 0.46 to 1.11, *P* = 0.14, *I*^*2*^: 47%). Publication bias assessment for this analysis showed a significant asymmetry in the funnel plot (Supplementary Fig. 2). However, Begg’s and Egger’s tests did not show any significant publication bias (*P* = 1.00 and 0.24, respectively).

Meta-analysis of neurological function assessment defined by mRS 0–1 was also performed and it was shown that patients on DOAC had significantly lower mRS 0–1, compared with controls (OR 0.68, 95% CI 0.61 to 0.76, *P* < 0.01, *I*^*2*^: 11%). This was the same for discharge (OR 0.72, 95% CI 0.64 to 0.81, *P* < 0.01, *I*^*2*^: 0%) and 90-day (OR 0.62, 95% CI 0.52 to 0.73, *P* < 0.01, *I*^*2*^: 0%) subgroups, as shown in Supplementary Fig. 3. This analysis had asymmetry in the funnel plot designed (Supplementary Fig. 4), while it was not significant in Begg’s and Egger’s tests (*P* = 0.73 and 0.43, respectively).

In assessing all-cause mortality, overall, patients with recent use of DOAC had no significant difference rate of all-cause mortality (OR 1.02, 95% CI 0.68 to 1.52, *P* = 0.93, *I*^*2*^: 40%, Supplementary Fig. 5). Three studies compared in-hospital mortality between DOAC and non-DOAC groups [7, 22, 23], and one study reported 90-day mortality in these groups [20]. In line, in each of the in-hospital mortality and 90-day mortality analyses, no significant difference was observed (OR 0.99, 95% CI 0.61 to 1.61, *P* = 0.96, and OR 0.87, 95% CI 0.20 to 3.72, *P* = 0.85). The funnel plot for this analysis revealed asymmetry (Supplementary Fig. 6). While Egger’s test also revealed significant publication bias by small study effects (*P* = 0.049), Begg’s test was insignificant (*P* = 0.734).

### Meta-analysis of safety outcome

Regarding symptomatic ICH as the main measure of safety, there was no significant difference between patients on DOACs and controls (OR 0.98, 95% CI 0.69 to 1.39, *P* = 0.92). However, this was associated with moderate heterogeneity (*I*^*2*^: 46%). The forest plot for this meta-analysis is shown in Supplementary Fig. 7. The funnel plot for this analysis is shown in Supplementary Fig. 8 and there was no asymmetry in that plot. The *P* of 0.852 and 0.462 for Egger’s and Begg’s tests show the same finding.

Meta-analysis of any ICH was also performed and it was shown that there is no significant statistical difference between the groups (OR 1.15, 95% CI 0.94 to 1.40, *P* = 0.18, *I*^*2*^: 0%). The forest plot for this analysis is shown in Supplementary Fig. 9. The funnel plot did not show any asymmetry (Supplementary Fig. 10); moreover, Begg’s and Egger’s tests did not reveal publication bias (*P* = 1.00 and 0.707, respectively).

## Discussion

Recent investigations showed that the rate of IVT was significantly lower in patients using DOACs than in controls, suggesting the physicians’ hesitance to use IVT in this therapeutic setting [24]. In this study, we did a systematic review and used a random effect model to conduct a meta-analysis to assess the mortality, functional neurological outcomes, and risk of sICH occurrence in patients with ischemic stroke who underwent IVT with and without a history of DOAC use and we discovered that the use of intravenous thrombolysis among patients receiving DOACs before stroke appeared to be reasonably well tolerated, might be beneficial, and not associated with increased risk of symptomatic intracranial hemorrhage.

In patients with nonvalvular AF, DOACs are now the treatment of choice for both primary and secondary prevention of ischemic stroke and systemic embolism [25]. However, while the rate of ischemic stroke has remained virtually stable, the relative risk of hemorrhagic strokes has been reduced by half by utilizing these medications in comparison to warfarin [26]. Once a patient experiences an ischemic stroke, the quick uptake of DOACs in clinical practice creates significant issues. DOACs directly affect factor X or thrombin in the coagulation cascade [27]. The risk of serious bleeding problems during stroke thrombolysis may be affected by these medications. The use of intravenous rt-PA in these individuals, however, has not been well investigated [22].

When using alteplase, hemorrhagic consequences are a big risk [28]. Because the plasma levels of DOACs peak 2 to 4 h or so after oral intake and hemorrhagic events are apparently associated with high-peak levels of DOACs, patients who get alteplase within 4 h of the previous dosage of DOACs may be at significant risk of hemorrhagic complications [20]. Twelve hours, or roughly half of a DOAC’s half-life, is another significant time interval following the last intake of a DOAC [20].

The difference in baseline characteristics of patients using IVT, including pharmacological interactions and unique genetic predispositions, limits the ability to determine the coagulation state by relying just on the duration since the last DOAC was taken [29]. Regarding the security of assessing DOAC concentrations before thrombolysis, significant prospective data are lacking, and it is unclear what the ideal threshold is below which intravenous thrombolysis can be carried out safely [30, 31].

Despite the fact that laboratory-based DOAC medication monitoring may be carried out and findings quickly obtained at specialized centers [32], this strategy does not appear to be practical for lower-volume, rural hospitals. Although it is currently not generally accessible for DOACs, point-of-care testing may provide a potential remedy. Prehospital triage at the dispatch center should start with asking about oral anticoagulant therapy if quick testing is not available locally [26]. If it is, a straight transfer to facilities with available test capacity would be initiated, reducing the time required for reperfusion therapy [26].

Current AHA/ASA guidelines state that rt-PA is contraindicated in patients taking DOACs unless the time since last intake is > 48 h or sensitive laboratory tests are normal and recommend IVT in DOAC-treated patients if there is no anticoagulant effect at the time of IVT application [22, 33]. It is important to note that relatively few of the instances that have been reported thus far had a final DOAC intake of more than 24 h. According to the elimination half-lives of dabigatran (12–14 h), rivaroxaban (5–9 h), and apixaban (12 h), patients with normal renal function can anticipate normal coagulation 24 h after last consumption (i.e., 2 half-lives) [22]. Also, INR b 1.7 has been defined as a cut-off value for IVT based on large cohorts [34].

The European Heart Rhythm Association (EHRA) guidelines currently remove dabigatran from the general recommendation that recommends IVT more than 24 h after the last DOAC consumption since idarucizumab permits reversal of the anticoagulant effect of dabigatran within minutes of delivery [33]. Mechanical endovascular reperfusion (MeR) is another treatment option in patients with acute ischemic stroke and is advised in DOAC-treated acute ischemic stroke patients with major vascular blockage, according to current recommendations [35, 36]. Despite the fact that recommendations differ amongst guidelines, the majority of them, including a current expert opinion, advise MeR without preceding IVT in patients who have received dabigatran treatment [33].

According to Shahjouie et al. [37], if we took into account all reports of IVT in acute ischemic stroke patients treated with DOACs, there was a higher probability of hemorrhagic transformation and early mortality in patients who got the antidote. This outcome was achieved despite the patients without prior reversal agent treatment having greater rates of concomitant conditions (diabetes mellitus, hypertension, hyperlipidemia, past stroke/TIA, and coronary artery disease) [37].

Andexanet alfa, a particular antidote for anti-Xa inhibitors, is another reversal medication that has been given permission for use in American patients taking rivaroxaban and apixaban [38]. There is a lack of information regarding the IVT’s clinical results in acute ischemic stroke (AIS) patients who received andexanet alfa. Anticoagulant reversal medications are also frequently unavailable and expensive [37].

Our study demonstrated that patients on DOACs had no increased risk of ICH and bleeding with no difference in mortality as well. However, there was an increased risk of functional independence in the DOAC group. One explanation for that could be the observational nature of the included studies and the lack of randomized controlled trials. As adjusted analysis by Kam et al. [7] showed, after adjustment for several baseline characteristics, patients with DOAC use tend to have better neurological outcomes as well (mRS 0–2). In other words, the presence of several comorbidities at baseline in patients on DOAC could be the reason for our meta-analysis finding. Patients taking DOACs fared better than those not taking any oral anticoagulants before a stroke in terms of going home after discharge and being ambulatory [22]. On the other hand, there are a number of potential explanations for the possible improvement of functional results in patients using DOACs. Patients who had recently taken DOACs could have low serum drug concentrations rather than none at the time of alteplase treatment. This could be due to slow metabolism in some patients whose last dose was taken more than 48 h prior to hospital admission or ineffective dosing in other patients whose last dose was taken within 48 h and who then experienced breakthrough ischemic strokes. Therefore, low concentrations of DOACs may enhance alteplase’s therapeutic benefits in recanalizing the target occlusion without unnecessarily escalating bleeding side effects. Another possibility might result from variations in the target occlusion composition between patient groups. The target occlusion in the DOAC population, when AF predominated as the stroke etiology, is often an embolism from a detached thrombus coming from the heart. The target occlusion’s composition may be more varied and occasionally involve admixed atherosclerosis and supervening thrombosis in the non-DOAC population. In comparison to vessels with residual atherosclerosis that are more likely to re-occlude, recanalization after alteplase may be more durable in relatively normal recipient vasculature from which thrombi have been removed [39]. Additionally, it is likely that these findings represent unmeasured or residual confounding [7].

According to previous studies, there might be three explanations for the lower ICH risk on DOAC, despite the fact that this was not observed in our study. First, neither factor VII nor VIIa plasma concentrations are impacted by DOAC. Warfarin, on the other hand, blocks the synthesis of factor VII. Second, compared to VKAs, DOAC has a smaller impact on post-ischemic blood-brain barrier permeability [40]. Third, DOAC lessens the activation of matrix metalloproteinase, which in turn lessens neurovascular dissociation [34].

The lower risk of ICH associated with DOACs may be especially important for patients with greater cerebral infarcts, older patients, and patients with other bleeding risk factors, such as cerebral microbleeds [41].

As far as we are aware, there have already been two meta-analyses on the subject of the security of endovascular therapy in DOAC-anticoagulated patients. According to the Kurowski et al. meta-analysis [42], patients who are on therapeutic anticoagulation had a similar rate of sICH as those who are not taking anticoagulants. The results of patients who took DOACs and VKA combined were given in this review [42]. Shahjouie et al. performed a meta-analysis of the reports that were available on the safety of IVT among AIS pretreated with DOACs in the 48 h or less prior to the administration of tPA bolus and in the absence of reversal agents. Their findings showed that the prior use of DOACs did not appear to increase the risk of symptomatic intracerebral hemorrhage in some AIS patients who were given IVT [37].

Diabetes and chronic kidney disease were present in older patients with symptoms of ICH, which are risk factors for developing ICH after using alteplase [20]. Additionally, Suzuki et al. demonstrated that increased systolic blood pressure and blood glucose at admission were independent risk factors for ICH after reperfusion therapy in DOAC patients [34], concurring with other reports [43, 44]. Several potentially causative factors, including heart rate regulation, cardiac output, and adjunctive therapies, may alter the severity of strokes either directly or by affecting thrombus size. If recurrent stroke occurrences from randomized controlled trials were pooled, it could be possible to determine if DOACs genuinely lessen stroke severity by reducing the rate of large vessel occlusion in comparison to VKA [24].

We are aware of certain limitations of this study. First of all, a potential source of bias was introduced by the short number of papers included in the meta-analysis and the analysis’s limited inclusion of subgroups. Additionally, there is a higher chance of selection bias due to the observational cohort design (not randomized) of the included studies and the significant baseline differences between patients who recently consumed DOACs and controls. Another significant flaw is that we were unable to pinpoint the precise cutoff for the last DOAC dose, which makes it difficult to draw a direct connection between the risk of bleeding and the date of the last DOAC intake. Finally, because Asians (lower dose) and other centers throughout the world submitted data, the IVT dose administered varied somewhat between included trials.

## Conclusions

According to the findings of this meta-analysis, the use of IVT for the treatment of ischemic stroke in a subset of patients taking DOACs was not associated with a significant increase in all-cause mortality when compared to patients who were not taking DOACs, and no remarkable differences in 90-days functional neurological outcome (mRS 0-2) were observed in subgroup analysis, and the rate of symptomatic ICH was nearly equal in both groups.

### Electronic supplementary material

Below is the link to the electronic supplementary material.

Supplementary Material 1

## Data Availability

Not applicable.
